# Experiences of women with cervical dysplasia and associated diagnoses using electronic cigarettes for smoking substitution

**DOI:** 10.1111/hex.12897

**Published:** 2019-04-21

**Authors:** Shirley A. James, Marshall K. Cheney, Katie M. Smith, Laura A. Beebe

**Affiliations:** ^1^ Department of Biostatistics and Epidemiology, College of Public Health University of Oklahoma Health Sciences Center Oklahoma City Oklahoma; ^2^ Department of Health and Exercise Science University of Oklahoma Norman Oklahoma; ^3^ University of Oklahoma Health Sciences Center Oklahoma City Oklahoma

**Keywords:** cervical dysplasia, E‐cigs, electronic cigarettes, smoking cessation

## Abstract

**Introduction:**

The aim of this qualitative study was to describe the motivation and experiences of women with cervical dysplasia and associated diagnoses who used electronic cigarettes (ECs) to reduce the number of cigarettes they smoked.

**Methods:**

Qualitative interviews were conducted with 26 women aged 18‐65 years with cervical dysplasia and associated diagnoses who smoked at least three cigarettes daily for the past year or more and who enrolled in an intervention designed to substitute regular cigarettes with ECs. At the 12‐week follow‐up, patients were contacted by telephone. Semi‐structured interviews were recorded, then transcribed, coded and analysed for themes.

**Results:**

When confronted with a new diagnosis associated with smoking, women in this study were eager to try ECs to help them reduce their intake of cigarettes. Women reported that physical cues similar to smoking, delivery of nicotine sufficient to assist with smoking reduction and the security of having the device available to use in instances where temptations to smoke may occur were all positive experiences in trying the device. Other women in the study reported negative experiences, such as a lack of sufficient nicotine to eliminate cravings, heaviness of the device and the need to keep it charged. Depression, nicotine addiction and habit were factors that made it difficult to decrease cigarette consumption.

**Conclusions:**

Findings suggest that ECs may help with smoking substitution in patients who must reduce smoking due to medical conditions or diagnoses.

## 1 INTRODUCTION

One in five women with human papillomavirus (HPV) 16 or 18 will develop cervical dysplasia, a condition in which precancerous cells proliferate on the surface of the uterus.^1^ Sociodemographic variables including younger age, unmarried status, being a member of a racial minority, poverty and low education are associated with testing positive for HPV.^2^ While the presence of HPV is independent of smoking status, smoking plays a critical role in the progression of HPV to cervical cancer.^3^, ^4^, ^5^ Smoking cessation is a critical intervention for all women diagnosed with HPV or cervical dysplasia who smoke.^3^, ^4^, ^5^ Because continued smoking significantly increases the risk of all‐cause and cancer‐specific mortality, smoking cessation is also important after cervical cancer diagnosis.^6^ To date, only one study has been reported using smoking cessation in this population. Santosa and associates counselled women with cervical dysplasia to stop smoking using the five “As”: Ask, Advise, Assess, Assist, and Arrange follow‐up.^7^ This study highlighted the importance of counselling, as 32% of their 125 subjects quit smoking with no other intervention. Because available research is limited, our understanding of cessation‐related barriers and motivators for this high‐risk population is incomplete.

Evidence‐based smoking cessation strategies include nicotine replacement therapy (NRT), prescription medications such as varenicline (Chantix or Champix) and bupropion (Wellbutrin or Zyban), and counselling, often used in combination.^8^ While these aids help smokers quit, they have low appeal and satisfaction.^9^ A smoking alternative showing promise for smoking substitution or reduction is cigalikes, or electronic cigarettes (ECs) that look like regular cigarettes. ECs demonstrate promise for immediate smoking cessation or reduction because although they provide nicotine, which is an addictive component, they also provide the helpful hand to mouth cues of smoking, without the carbon monoxide and combustion that cause regular cigarettes to be most harmful.^10^

Since 2007, the United States has observed an increase in the sale and use of ECs, both for nicotine delivery and for nicotine reduction, or cessation purposes.^11^ Whether or not these devices are effective for smoking cessation is a question that remains unanswered. Results of two randomized controlled trials suggest that ECs may contribute to smoking cessation or at least be as effective as the nicotine patch.^12^, ^13^ In addition, qualitative studies can help clinicians and researchers understand why some smokers are able to successfully transition to ECs but others are not. Studies have been published with naive^14^, ^15^, ^16^ as well as experienced EC users,^17^, ^18^, ^19^, ^20^, ^21^, ^22^, ^23^ and the most common theme reported was the high level of satisfaction and acceptability of ECs.^14^, ^15^, ^18^, ^19^, ^22^, ^23^ Users stated the devices helped with smoking reduction or cessation^20^, ^22^, ^23^ were less costly than cigarettes,^21^ posed fewer health risks than smoking,^19^, ^21^, ^23^ lacked the negative smell of cigarettes^14^, ^21^, ^24^ and provided the positive physical cues of hand to mouth simulation.^14^, ^16^, ^17^, ^21^, ^22^, ^23^, ^24^ EC users also reported requiring less nicotine from ECs to satisfy their nicotine addiction, which led to lower nicotine dependence. Some EC users report nicotine withdrawal relief within five minutes of using the devices.^21^ Alternatively, EC users have also reported negative effects, stating they were not helpful for smoking cessation and caused a burning in the throat or a dry mouth.^16^, ^19^, ^22^, ^23^

The use of ECs for smoking substitution or reduction in groups of individuals receiving medical intervention or who are at an elevated risk for cancer has not been investigated. The aim of this qualitative study was to describe the motivation and experiences of women with cervical dysplasia and associated diagnoses who used electronic cigarettes to substitute for or reduce the number of cigarettes they smoked.

## 2 METHODS

This study was approved by the University of Oklahoma Health Sciences Center Institutional Review Board. Participants were women who had been diagnosed with cervical dysplasia, cervical cancer or lower genital tract dysplasia within the last month, and who enrolled in a research intervention designed to substitute regular cigarettes with electronic cigarettes.^25^ The physician in charge of the clinic determined medical eligibility for the study.

### 2.1 Participants

#### 2.1.1 Inclusion criteria

Inclusion criteria included current female smokers, aged 18‐65 years. All participants were diagnosed with and being medically treated for cervical dysplasia, cervical cancer or lower genital tract dysplasia by the doctor in charge of the clinic. Smokers were defined as those who in a typical week smoked an average of three or more cigarettes a day and had done so for at least the last year.

#### 2.1.2 Exclusion criteria

Exclusion criteria included patients unwilling to commit to a 6‐week intervention; with current diagnoses of or treatment for other cancer as ascertained from their medical record; and with a diagnosis of stroke, heart disease or high blood pressure not well controlled with medication as documented in their medical records. Additional exclusion criteria included women who were pregnant, ascertained via urinary analysis, lactating or planning pregnancy in the next 6 months, ascertained by their report, and currently using ECs or vaping systems.

### 2.2 Procedures

After an initial screen for eligibility, women were consented into the study. Women then sampled ECs and decided if they were willing to use them for a 6‐week intervention period designed to assist with smoking reduction/cessation. Women received two sessions of motivational interviewing, a Blu^®^ EC starter kit containing two batteries and a charger at the first session, and a 6‐week supply of nicotine cartridges, given according to manufacturers’ recommendations.

### 2.3 Measures

To assess smoking behaviours and EC use, women were asked, “In the last week, on average, how many cigarettes did you smoke each day?” If at follow‐up the answer was none, women were asked the date they smoked their last cigarette, “even a puff.” During follow‐up phone calls, women were also asked how many EC cartridges they had used. EC use was recorded as the total number of cartridges each woman used during the previous 6‐week period. This measurement of smoking behaviours and EC use was completed at baseline, at a 6‐week follow‐up and after an additional 6‐week period when intervention products were no longer supplied.

At the 12‐week follow‐up telephone call, women were interviewed about their experiences with ECs. Twenty‐six women participated in the interviews and were compensated with a $20 gift card at the 12‐week follow‐up to partially compensate for their time. Women were also able to keep their EC device, their charger and carrying case.

### 2.4 Qualitative interview coding

Semi‐structured telephone interviews were conducted at 12 weeks to determine women's motivation to reduce smoking, as well as their response to the ECs provided by the study to assist them in doing so. Interviews lasted 20‐45 minutes and were audio‐recorded, and then transcribed. Transcription was completed by one of the authors (SJ) and included verbatim transcription including nuances of the interviews not apparent from the actual wording of the conversation. Two of the authors (SJ and MC) then developed a detailed codebook, coded four transcripts together using NVivo (version 10; QSR International) and made revisions to the codebook. When satisfied with the initial codebook, these authors separately coded each interview. The two authors then reviewed the codes on a weekly basis until consensus was achieved on the core codes. Authors then sorted the codes into developing themes using the NVivo program for thematic maps. The two authors then refined the themes, assuring the data fit well into each theme without overlaps. Authors then reviewed the entire data set to assure consistency within the themes and were satisfied the coding fits the data well.

Following thematic analysis, the transcripts were reviewed again for additional supporting and disconfirming evidence of themes by the third author (LB). When all three authors met consensus on the main themes reached, quotes representing the themes were added to illustrate themes and subthemes with quotes identified by subject number.

## 3 RESULTS

Twenty‐six women with a mean age of 40 years completed follow‐up interviews. Twenty‐seven per cent did not finish high school, and 23% finished high school or had a general education diploma. Almost one in four (23%) had no insurance, and over a third (42%) had Medicaid only. Most women in the study (85%) had an annual income of <$25 000 (Table 1).

**Table 1:** Sociodemographic characteristics of participating patients (n = 26)

	Min	Max	Mean	Median	Standard deviation
Age	21	64	40.0	35	13.08
Number of years of smoking history	7	57	22.0	20	11.77
Race/Ethnicity					
White	18 (69.2%)				
African American	4 (15.4%)				
Native American	3 (11.5%)				
Hispanic	1 (3.8%)				
Annual Income					
<$25 000	22 (84.6%)				
$25 001‐$50 000	4 (15.4%)				
>$50 000	0				
Insurance Coverage					
Medicaid only	11 (42.3%)				
No insurance	6 (23.1%)				
Private insurance	5 (19.2%)				
Combination Medicare and Medicaid	4 (15.4%)				
Education					
<HS	7 (26.9%)				
HS graduate or GED only	6 (23.1%)				
Some college (no degree)	13 (50%)				
College degree	0				

### 3.1 Smoking and EC use at the 12‐week follow‐up

In a self‐report telephone interview, after their 6‐week trial of ECs for smoking cessation, followed by their 6‐week trial with no intervention, 17 of 26 women continued to use ECs. Eight women using ECs quit smoking cigarettes entirely, 11 reduced cigarette use by 50% or more, and seven reduced by <50%. Four women in the study no longer used nicotine of any kind (Table 2).

**Table 2:** Smoking and EC use at time of interview by reported EC experience (n = 26)

	All women (n = 26)	Women with a positive EC experience (n = 12)	Women with a negative EC experience (n = 4)	Women with a mixed EC experience (n = 10)
EC use				
Continued to use ECs	17	9	1	7
Cigarette use (Smoking)				
Quit smoking cigarettes	8	6	0	2
Reduced # of cigarettes per day by 50% or more	11	5	1	5
Reduced # of cigarettes per day by <50%	7	1	3	3

Overall, 12 women reported overwhelmingly positive experiences with ECs; four reported primarily negative experiences; and ten described a mixture of positive and negative experiences. At the 12‐week follow‐up, 75% of women with a positive EC experience continued to use them at the time of the interview. Similarly, 70% of women who reported mixed experience with ECs continued to use them. Among the four women who reported only negative experiences with the EC, none had quit smoking and only one continued to use ECs (Table 2).

### 3.2 Qualitative results and themes

**Figure 1:**
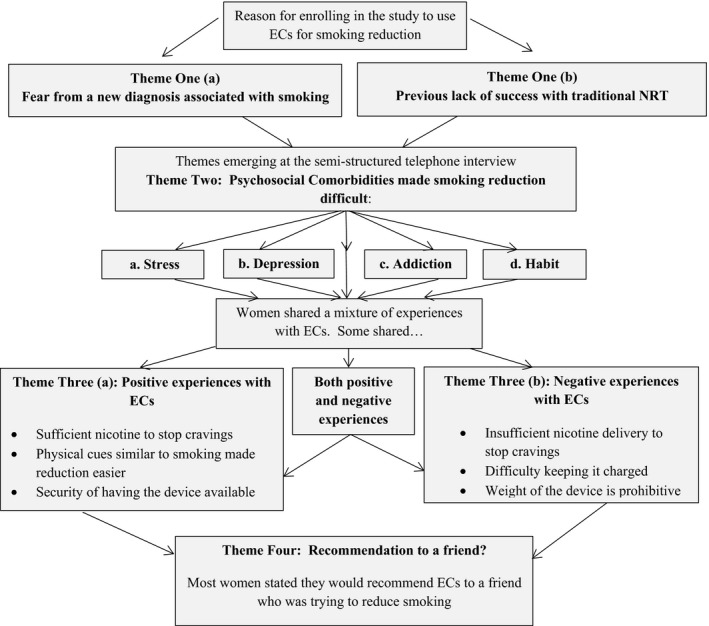
Flow diagram of themes emerging from this study

An overview of themes discussed by women in this study is displayed in Figure 1. Included are motivators for using ECs, problems women encountered during their smoking reduction attempts and whether their experience was positive, negative or mixed, and why.

#### 3.2.1 Theme one: Motivating variables

##### The impact of a new diagnosis

All 26 women in the study noted their biggest motivation for trying ECs for smoking reduction was their new diagnosis. Nine women noted that fear that their new diagnosis would develop into invasive cervical cancer made them especially motivated to reduce smoking. Before the study started, only five remembered having previously been told about the link between smoking and cervical dysplasia in women with HPV. Women said they appreciated staff who took the time to explain that association in a non‐judgemental way, and noted receiving information at the time of diagnosis heightened its impact.

The oncologist here had the biggest impact on me. Dr Smith told me to stop smoking in a way that was uh…..easy? It impacted me. Doctors that tell you to stop don't help you and try to shame you – you let that go in one ear and out the other. She just was able to tell me why and it made sense. (#11, reduced by 70%, still using ECs)

Staff explained the difference stopping smoking would make on my dysplasia. They really had an impact on my wanting to quit. When they told me it was connected to cervical cancer I was very surprised. I did not know that. It makes me want to quit all the more. (#20, reduced by 25%, still using ECs)

##### Lack of success with previous smoking cessation products

Sixteen of the 26 women had tried nicotine replacement therapy or another smoking cessation method in the past with limited or no success. Most women expressed burnout or frustration that these products had not worked for them. Two women noted the support they received during previous unsuccessful quit attempts ended prematurely and left them feeling deserted. As a result, all 26 women were enthusiastic to try ECs as a smoking substitute. Most had tried someone else's vaping device and were eager to see whether ECs could help them reduce or eliminate their smoking habits.

I have tried patches for 1 month. I also tried hypnosis about 25 years ago. It didn't work. Yes, I have tried about everything. I tried smoking cessation counseling with the quit line in 2011. It didn't work either. Probably because I have smoked so very long! It has been a really hard, hard habit to deal with. I had friends who had used the electronic cigarettes and that made an impression on me. I kind of….well you know, I was curious. You hear the advertisements and things of that nature and you get curious. Then you hear others say “I used it and it worked for me.” You get feedback like that and you are curious. I was excited to try it. (#9, reduced smoking by 60%, EC user)

P: In the past I tried Chantix (Varenicline). When I tried that for thirty days I had some success but then my insurance wouldn't pay anymore. I: How did that make you feel? P: Like I was a piece of crap to them. Like I was nothing. Like I was not important. It made me want to start smoking again. (#2 no longer smoking, no longer using ECs)

#### 3.2.2 Theme two: Psychosocial comorbidities

As women struggled to reduce smoking regular cigarettes and replace them with ECs, 14 mentioned stress, eight mentioned addiction, six mentioned depression, and five mentioned habit as difficulties they experienced.

##### Stress

Women experienced stress related to their new diagnosis and treatment, combined with the need to reduce smoking to improve their prognosis. When additional stressors were added to their lives from outside sources including family, friends, work or lack of income, many were especially tempted to revert back to previous smoking habits as a form of stress relief.

But I have been so stressed out too. Yes, physically, mentally, emotionally, and in every way. I know I have not been doing well. I really haven't. But stress is a real thing. Stressful times like these are when I guess we really test these products. (#25, reduced smoking by 75%, EC user)

But my life is so crazy. I have kids and grandkids at home and everyone seems to want something from me all the time and all at once. This was just not the time for me to stop smoking. I know I need to quit. I will keep trying. It will be a long haul. I thought it would go quickly at first, but it won't. (#10, reduced smoking by 65%, EC user)

…this diagnosis is not helping any. I have to always go to the doctor and I can't afford to. But I can't afford not to either, you know what I mean? If I don't go, I die of cancer. You can see why I can't quit smoking, right? I tried and tried to get enough nicotine that I didn't need a smoke, but I just couldn't do it. (#3, increased smoking, no longer using ECs)

##### Depression

After initial feelings of shock due to their new diagnosis, most women expressed some level of depression. This was especially true in a group of women struggling with smoking reduction who felt defeated that this was yet another failed smoking reduction attempt. This sense of failure seemed to permeate other areas of their lives.

Of course I want to quit smoking, everyone wants to quit smoking. Especially with my diagnosis. I just can't deal with it all right now. Even though I want to quit, I just can't try right now. I am just too depressed. (#3, increased smoking, not using ECs)

##### Addiction

When asked about barriers to smoking reduction, most women mentioned the addictive nature of cigarettes, which many blamed on cigarette companies. Many women struggling with smoking reduction expressed feeling overwhelmed by their addiction to smoking.

I think smoking is way far more addicting than other things out there. It's just so addictive. I think it is an addiction that I just can't overcome. (#18 reduced smoking by 43%, not using ECs)

##### Habit

Although each woman expressed the desire to reduce or quit smoking cigarettes, most stated their well‐established smoking habits constituted a huge barrier.

“I wish I could go longer with just a couple of puffs, but that is just asking too much right now. I know I will and can quit with the electronic cigarette.” Habit. Habit. Habit. Someday I would like to hang a big sign that says “I am now a non‐smoker!” (#9 reduced smoking by 60%, EC user)

I think that a lot of times whenever we grab up a cigarette it's so we have something in our hands and in our mouths you know…that is why we grab one up. Because of that habit. The Blu helps with that. (#21, reduced smoking by 60%, still using ECs)

#### 3.2.3 Theme three: Women's experiences with ECs

##### Positive experiences with EC use

Most women stated that the nicotine they received was sufficient to satisfy their cravings. The most common attribute reported by these women was the important physical cues the EC provided, including the feel of the device in their fingers, the hand to mouth motion, the ability to inhale and exhale the vapour, and the ability to play with the device. Women who had substituted most or all of their cigarettes with ECs felt secure knowing they could carry the device with them. They noted it was available when they “just had to have a smoke,” especially in nightclubs, bars and parties where others were smoking.

The e‐cigarette helped me to stop. Period! It was the same rush as a cigarette. It calmed me down. It kept me from wanting to smoke. It got rid of the cravings. It helped me so much because with a puff it kept me from wanting a whole cigarette. I use it whenever I go out with my friends to the bar. They are smoking ‐ killing themselves, and I pull out my e‐cigarette if I need it and I take some draws on it and I totally don't have to smoke. It's great. (#2, quit smoking, EC user)

I loved the e‐cigarette. I was able to use it when I just “had to have a smoke.” It was easy to use and it looked and felt like a regular cigarette. I could bring it to my mouth and inhale. It met all my “habits” that I had. I loved using it. I don't think I will ever start smoking again. (#13, quit smoking, EC user)

##### Negative experiences with EC use

In direct opposition to women who reported that ECs provided enough nicotine to eliminate their cravings, other women noted that the device did not deliver enough nicotine to help them with withdrawal symptoms. Several noted ECs were too different from regular cigarettes to provide the cues they missed from smoking. Others indicated the weight of the device and the need to keep it charged made its use unmanageable.

It is heavier than an actual cigarette. I don't think I will keep using it because it is heavier and I worry about wrinkles. (#15, no smoking reduction, not using ECs)

I don't use the electronic cigarette at all anymore ‐ It's not that I dislike it – I think I am just lazy and I don't get it charged – it is too much work. (#18, reduced smoking by 43%, not using ECs).

You don't get all your nicotine. But they do help. It was not quite enough nicotine because I smoke so heavy. It would be hard to replace all the nicotine. It is hard sometimes because you have to puff really hard to get it to work. It is easy to use, but you have to puff really hard. (#21, reduced smoking by 60%, EC user)

#### 3.2.4 Theme Four: Women's recommendations

Most women in this study (18/26) said they would recommend ECs to a friend who was trying to reduce or stop smoking. This included women who themselves had negative experiences with EC use.

Yes, I would tell others to use this product because it helped me absolutely quit smoking. I don't smoke at all anymore. And I am pretty confident I won't start again. (#4, quit smoking, EC user)

I: Would you recommend this product for others to use? S: Of course. I: Why, since it didn't work for you? S: It might work for them. Everybody's different. This didn't make it more difficult for me to quit, it just didn't help me quit. (#8 decreased smoking by 25%—not using ECs)

## 4 DISCUSSION

The results of this qualitative study are consistent with others suggesting ECs can provide helpful physical cues, as well as nicotine substitution, for smokers attempting to reduce their cigarette intake or quit smoking altogether.^14^, ^16^, ^17^, ^21^, ^22^ The population in this study was unique, because added to their desire to reduce cigarette consumption for general health reasons, was the desire to reduce cigarette consumption to prevent invasive cervical cancer. Women had used a wide variety of smoking cessation aids, ranging from traditional nicotine replacement therapy to hypnosis with little success. Some, but not all women, found ECs to be more helpful. Consistent with reported literature, several women in this study reported that the EC device did not deliver enough nicotine to reduce cravings, was too heavy and required charging, all making it unappealing for smoking reduction.^16^, ^19^, ^22^, ^23^ At the point of discontent, these women may have been more successful if they had been allowed to try a different aid.^8^ Examples include the nicotine inhaler, or a second‐generation vaping system that would provide larger doses of nicotine more quickly, with a wider variety of flavours and nicotine strengths.^26^

Especially provocative for women with positive experiences with ECs was the presence of strong physical cues including bringing the device from hand to mouth, drawing the vapour into the lungs and holding the device between the fingers, all well documented in other studies.^14^, ^16^, ^17^, ^21^ Unique to this population of women with cervical dysplasia was the need for the security the device provided. Women noted the device was available “when they needed it,” or “when they had the urge to smoke,” particularly helpful in social situations when others around them were smoking, or when stressful life situations made it appealing to return to previous smoking habits. Zhou and associates found providing a replacement for smoking cues was especially important for smoking cessation over time, noting relapse was associated with exposure to smoking cues, cravings, withdrawal symptoms or lack of a smoking cessation aid,^27^ problems resolved using ECs.

When these women with cervical dysplasia struggled with cigarette reduction, they most often cited habit, addiction, depression and stress as factors making it difficult. Habit and addiction are not surprising, given these women's smoking histories, which ranged from seven to fifty‐seven years. Several women in the study expressed defeat and resignation at yet another unsuccessful smoking reduction attempt, and their interviews reflected strong feelings of failure, which could derail future quit attempts. When these women experienced overwhelming stress from life situations, it was not realistic to expect them to additionally reduce or quit smoking, because smoking constituted their coping mechanism for stress.^28^ The point at which they begin to resort to old habits of using smoking for stress relief is the time when additional support, including depression or mental health counselling, is most important. Several women also noted the importance of long‐term follow‐up support. Sohl, Birdee & Elam advocate motivational interviewing, as well as eliciting the aid of a life or health coach to empower continued behavioural change.^29^ Without long‐term support, women may experience feelings of desertion that several mentioned during previous unsuccessful quit attempts when support had been withdrawn too soon.

Although many women in this study did not completely substitute their cigarettes with ECs, any reduction in the number of cigarettes smoked daily represents success. In a qualitative study, Hughes & Carpenter concluded that smoking reduction increased the probability of future cessation.^30^ The potential value of EC use for smoking reduction, even in women not completely successful, was best represented by women's willingness to recommend them to a friend who was trying to quit smoking.

It is difficult to determine whether the information gained from these women's experiences will be generalizable to other women with cervical dysplasia or to patients with other medical conditions associated with smoking and whose prognosis depends on smoking reduction or cessation. Only four women in this study had exclusively negative experiences with ECs for smoking substitution, making it difficult to determine whether a complete description of possible negative experiences was accurately represented. It is important to note that while using ECs is a harm reduction technique, ECs still provide nicotine, an addictive substance. Additionally, this study only examined experiences with cigalike devices. Smokers are turning to second‐ or third‐generation vaping devices for more successful smoking reduction and cessation,^17^, ^31^ due to improved levels of nicotine delivery and satisfaction.^32^ In future studies, these devices should be included as a substitution/reduction method.

## 5 CONCLUSION

Twenty‐six women with cervical dysplasia and associated diagnoses were given the opportunity to try smoking reduction using ECs and were then given the opportunity to share their experiences through qualitative interviews. Women in this study believed that the potential medical complications that could arise if they continued to smoke were severe enough to warrant a serious attempt to reduce or eliminate their cigarette consumption with the help of ECs.

Despite the fact that ECs have not been proven to be a safe long‐term alternative to smoking, this study adds to the growing body of research that suggests that ECs can provide adequate nicotine delivery for the alleviation of cravings and the strong physical cues smokers miss when trying to cut down on smoking. These benefits may be relevant to other patients with medical conditions in which the danger of smoking is magnified.

## CONFLICT OF INTEREST

James, Cheney, Smith and Beebe have no financial disclosures.

## DATA AVAILABILITY STATEMENT

The data that support the findings of this study are available from the corresponding author upon reasonable request
